# Methotrexate, vinblastine, doxorubicin and cisplatin combination regimen as salvage chemotherapy for patients with advanced or metastatic transitional cell carcinoma after failure of gemcitabine and cisplatin chemotherapy

**DOI:** 10.1038/sj.bjc.6604113

**Published:** 2007-12-18

**Authors:** K S Han, J Y Joung, T S Kim, I G Jeong, H K Seo, J Chung, K H Lee

**Affiliations:** 1 Urologic Oncology Clinic, Center for Specific Organs Cancer, National Cancer Center, Goyang, Korea

**Keywords:** transitional cell carcinoma, chemotherapy, cisplatin, metastasis

## Abstract

We investigated the safety and efficacy of a methotrexate, vinblastine, doxorubicin and cisplatin (M-VAC) combination regimen as second-line chemotherapy for patients with advanced or metastatic transitional cell carcinoma who failed first-line gemcitabine and cisplatin (GC) chemotherapy. Thirty patients who had progressed or relapsed after GC chemotherapy as first-line treatment were enrolled in this study. The major toxicities were neutropaenia and thrombocytopaenia. A grade 3 or 4 neutropaenia occurred in 19 (63.3%) and a grade 3 or 4 thrombocytopaenia developed in nine patients (30.0%). There were no life-threatening complications during the study. The overall response was 30%. A complete response was achieved in two patients (6.7%) and a partial response in seven (23.3%). The overall disease control rate was 50%. Seven out of 16 patients who had responded previously to GC responded to M-VAC, while 2 out of 14 who had not responded to GC responded to M-VAC. The median response duration was 3.9 months and the median progression-free survival was 5.3 months. The median overall survival was 10.9 months. M-VAC showed encouraging efficacy and reversible toxicities in patients who had progressed after GC chemotherapy and, especially, M-VAC appears to be a reasonable option as a sequential treatment regimen in patients who responded previously to GC chemotherapy.

Urothelial carcinoma is the second most common genitourinary malignancy. Since advanced urothelial carcinoma is sensitive to chemotherapeutic agents, systemic combination chemotherapy has been the standard treatment that can result in long-term survival in some patients with advanced or metastatic transitional cell carcinoma. The most commonly used regimens are the methotrexate, vinblastine, doxorubicin and cisplatin (M-VAC) combination or the gemcitabine and cisplatin (GC) combination regimen (Bamias *et al*, 2006; Garcia and Dreicer, 2006; Pectasides *et al*, 2007). The M-VAC regimen was introduced at the Memorial Sloan-Kettering hospital in the mid-1980s and it improved overall survival to a median slightly in excess of 12 months (Sternberg *et al*, 1988; Culine, 2002). Thereafter, a GC regimen was developed and, when it was compared with M-VAC, it showed comparable efficacy with reduced side effects (Moore *et al*, 1999; von der Maase *et al*, 1999, 2000; Kaufman *et al*, 2000). Recently published long-term results confirmed a similar 5-year survival and progression-free survival rates for GC and M-VAC (von der Maase *et al*, 2005). On the basis of these results, M-VAC or GC has become the standard first-line treatment for patients with advanced transitional cell carcinoma.

However, a dismal prognosis is predicted in patients whose disease progresses after first-line chemotherapy. Therefore, safe and effective second-line chemotherapy regimens are needed for patients who have a disease relapse after initial chemotherapy treatment. Currently, there is limited information on additional treatment of patients who fail first-line platinum-based chemotherapy. Taxanes, gemcitabine and ifosfamide have shown promising results after failure of first-line chemotherapy (Tu *et al*, 1995; McCaffrey *et al*, 1997; Witte *et al*, 1997; Sweeney *et al*, 1999; Krege *et al*, 2001; Meluch *et al*, 2001; Sternberg *et al*, 2001; Bellmunt *et al*, 2002; Vaughn *et al*, 2002). However, the response rates to these regimens have been variable and durable remissions have been limited.

Although GC is widely considered as the new standard for first-line treatment in patients with advanced bladder cancer, in several countries, there is no data on salvage chemotherapy after failure of GC. Moreover, the role of M-VAC as second-line treatment after failure of GC is unknown. We therefore investigated the safety and efficacy of the M-VAC regimen as salvage chemotherapy for the patients in whom first-line GC chemotherapy failed.

## PATIENTS AND METHODS

### Patient eligibility

Patients with histologically confirmed advanced or metastatic transitional cell carcinoma were eligible to participate in this study. All patients had evidence of disease progression or relapse after GC chemotherapy as first-line treatment. For GC chemotherapy, gemcitabine was given at a dose of 1000 mg m^−2^ on days 1, 8 and 15, and cisplatin was given at a dose of 70 mg m^−2^ on day 2. Adequate organ function was required and an Eastern Cooperative Oncology Group performance status of 0–2, an absolute granulocyte count ⩾1500 mm^−3^, platelet count ⩾100 000 mm^−3^, serum creatinine ⩽1.5 mg dl^−1^, creatinine clearance ⩾50 ml min^−1^ and a serum bilirubin ⩽2 mg dl^−1^ were required for the treatment. The exclusion criteria included the presence of brain metastases or persistent toxicity from previous GC chemotherapy. Informed consent was obtained before entry into the study. The study was conducted in accordance with the Declaration of Helsinki Principle and Good Clinical Practice and was approved by an independent ethics committee.

### Treatment schedule

The patients received methotrexate 30 mg m^−2^ on days 1, 15 and 22; vinblastine 3 mg m^−2^ on days 2, 15 and 22; doxorubicin 30 mg m^−2^ on day 2; and cisplatin 70 mg m^−2^ on day 2. The cycles were repeated every 28 days. The patient response was evaluated every three cycles. An additional three cycles with same regimen were provided to patients with no progression at the response evaluation. A third-line chemotherapy regimen was initiated in patients who had progressed if the patients had a good performance status and adequate organ function.

### Response and toxicity assessment

All patients were evaluated for their response to treatment after completing three cycles except for those cases with symptomatic progression. Early evaluations were performed in patients with clinical evidence of progressive disease (PD). Patient response was evaluated according to the Response Evaluation Criteria in Solid Tumors (Therasse *et al*, 2000). A complete response (CR) was defined as the disappearance of all clinical and radiological signs of target lesions. A partial response (PR) was a ⩾30% decrease in the overall sum of the diameter of the target lesions; a PD was a ⩾20% increase in the overall sum of the diameter of the target lesions; and stable disease was neither a sufficient shrinkage to qualify for PR nor sufficient increase to qualify for PD. In cases with a PR or CR, a second assessment 4 weeks later was required for confirmation of the response. The toxicity was graded according to the National Cancer Institute's common toxicity criteria version 2.0. Patients who received at least one dose of M-VAC were assessed for toxicity.

### Statistical considerations

The duration of response was defined as the time from the first objective status assessment of response to the initial date of a documented progression. The progression-free survival was defined as the time from study entry to the initial date of evidence of progression, death or loss to follow-up. The progression-free survival of patients alive without progression was recorded at the time of the last follow-up evaluation. The overall survival was defined as the interval between the date of study entry to death or the last follow-up evaluation. The progression-free survival and the overall survival were estimated using the Kaplan–Meier method. The analyses were performed using 5% as the level of significance.

## RESULTS

### Patient characteristics

Between May 2002 and December 2006, 30 patients were enrolled in this study. The baseline characteristics of the patients are shown in Table 1. The median age was 64 years (range, 38–79). There were 24 men and 6 women. Fourteen patients had primary tumours. Nineteen patients had visceral metastases and 11 patients had regional lymph-node metastases only.

For first-line GC chemotherapy, the median number of cycles was five (range, 2–9) and the response rate was 53.3%. There were no interrupted schedules or dose adjustments due to persistent toxicities. All patients received at least three cycles of GC except for one patient. In one patient, GC chemotherapy was discontinued during a second cycle because acute renal failure developed due to progression of pelvic lymph node metastases.

After disease progression or relapse was confirmed, the patients received the M-VAC regimen. The median treatment-free interval between GC and M-VAC was 2.5 months (range, 0.5–20.4) and the median number of cycles for the M-VAC regimen was three (range, 1–12).

### Toxicity

All enrolled patients were evaluated for toxicity (Table 2). The treatment was generally well-tolerated. The major haematological toxicities were neutropaenia and thrombocytopaenia. A grade 3 or 4 neutropaenia occurred in 19 patients (63.3%); they received granulocyte colony-stimulating factor until their neutrophil counts were restored. A grade 3 or 4 thrombocytopaenia developed in nine patients (30.0%); there was no haemorrhagic event due to the thrombocytopaenia. Grade 3 or 4 anaemia developed less frequently and was identified in five patients (16.7%). The major nonhaematologic toxicities were alopecia and mucositis. Grade 2 alopecia developed in 14 patients (46.7%) and grade 3 or 4 mucositis in four (13.3%); most nonhaematologic toxicities were confined to grade 1 or 2 (Table 2). All toxicities were reversible and no life-threatening complications were observed during the study.

Omission of methotrexate and vinblastine on day 15 and/or 22 or delay of a subsequent cycle occurred in cases with persistent severe haematological toxicities despite appropriate management. Methotrexate and vinblastine on day 15 or 22 were omitted in 23 patients. The median for an omitted scheduled cycle was one per patient (range, 0–16). The schedules on day 15 or 22 were delayed in 11 patients. The median number of delayed schedules was zero (range, 0–3); all patients could receive scheduled treatments after 1 week. A subsequent cycle was delayed for 1 week in one patient.

### Response rates and survival

The response to treatment was assessed in all registered patients. The overall response rate was 30%. A CR was achieved in two patients (6.7%) and a PR in seven (23.3%). Stable disease was observed in six patients (20.0%). The overall disease control rate was 50%. The characteristics of the responders are listed in Table 3. Seven out of 16 patients who had responded previously to GC responded to M-VAC, while 2 out of 14 who had not responded to GC responded to M-VAC. A response was observed in 4 out of 11 (36.4%) patients with only lymph node metastases and in 5 out of 19 (26.3%) patients with visceral metastases.

**Figure 1:**
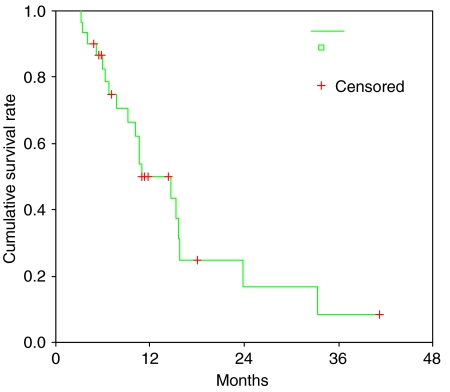
Kaplan–Meier survival curves of overall survival.

The median response duration was 3.9 months (95% CI: 3.7–4.1) and the median progression-free survival was 5.3 months (95% CI: 3.1–7.5). A majority of patients finally had disease progression; only three patients remained with no disease progression at the last follow-up evaluation. Twenty patients died and 10 patients were still alive at the last follow-up. The median overall survival was 10.9 months (95% CI: 5.5–16.3). The actuarial 1- and 2-year survival rates were 49.8 and 16.6%, respectively (Figure 1).

## DISCUSSION

M-VAC is the most effective regimen as first-line treatment for transitional cell carcinoma of the urothelial tract but the role of M-VAC as second-line chemotherapy has never been studied although a variety of other agents have been investigated. Since GC chemotherapy was introduced as first-line chemotherapy for urothelial carcinoma, the role of the M-VAC regimen after GC remains to be defined. The results from our study showed that M-VAC chemotherapy was effective for urothelial carcinoma progression following first-line GC chemotherapy. M-VAC appears to be effective in patients who become less sensitive to the synergistic effects of gemcitabine and cisplatin.

In our study, the M-VAC regimen had a response rate of 30% in patients with disease progression after GC failure. There may be several explanations for this result. Cisplatin is one of the most potent chemotherapeutic agents for advanced transitional cell carcinoma and the failure of GC treatment does not imply a cisplatin-refractory status. Cisplatin may still be active in patients who failed prior GC chemotherapy. In addition, patients who fail the GC protocol may be sensitive to other potent agents such as methotrexate, doxorubicin and vinblastine. Our favourable results suggest that M-VAC is another option in patients with advanced transitional cell carcinoma who failed first-line GC treatment. On the other hand, we found a trend for a correlation between the response to the first-line GC treatment and the response to second-line M-VAC. Seven out of 16 patients (43.8%) who had responded to the prior GC therapy responded to the second-line M-VAC chemotherapy, while only 2 out of 14 (14.3%) who did not respond to GC therapy responded to M-VAC. These findings suggest the possibility that the response to the second-line M-VAC can be predicted from the response to the first-line GC chemotherapy. The difference of the response rates to second-line M-VAC between those who responded to GC and those who did not respond to GC treatment was not statistically significant but the absence of significance might have been due to the small number of patients studied. Additional trials are needed to confirm whether the response to first-line GC treatment can be a significant predictor of the response to second-line M-VAC.

Most patients enrolled in this study had good renal function and performance status. Our favourable results might have been affected by these factors. It is reported (de Wit, 2003) that an estimated one-third of patients with advanced urothelial carcinoma are medically unfit for cisplatin-based chemotherapy , and in accordance with this, in clinical practice, there is frequently a significant deterioration of the performance status or renal function in patients with advanced urothelial carcinoma; this is most frequently observed in patients with first-line treatment failure and disease progression. This makes enrolment of patients into clinical studies or the administration of systemic chemotherapy outside of the context of a clinical trial difficult (Bamias *et al*, 2006; Garcia and Dreicer, 2006; Pectasides *et al*, 2007). Substitution of new agents for cisplatin in two-drug or three-drug combinations is required in these patients. Carboplatin can be a good alternative to cisplatin because it is less nephrotoxic (de Wit, 2003). Phase II trials of carboplatin, as a substitution for cisplatin in first-line chemotherapy regimens, demonstrated that carboplatin-based therapy was less effective than cisplatin-based therapy. However, there is few data on carboplatin as a substitution for cisplatin-based therapy in second-line chemotherapy (Petrioli *et al*, 1996; Bellmunt *et al*, 1997). Randomised trials with carboplatin-based regimens in patients who failed first-line platinum-based chemotherapy and development of new combinations consisting of safer and more effective agents is needed for the treatment of patients with depleted marrow reserves and impaired renal function after the failure of first-line chemotherapy.

Over the past 20 years, a relatively large number of agents have been evaluated for second-line chemotherapy. The agents studied included ifosfamide, taxanes, gemcitabine, oxaliplatin, vinflunine and pemetrexed. However, a review of the recent literature, on phase II trials in this population, confirms that there are very limited options for patients who have been previously treated with GC, M-VAC or CMV combinations (Culine *et al*, 2006). Taxanes are widely used as a second-line regimen in patients with cisplatin-refractory urothelial carcinoma. The taxanes (paclitaxel and docetaxel) have provided objective response rates of 10–13% with response durations of 3.0–7.4 months (McCaffrey *et al*, 1997; Vaughn *et al*, 2002). Combinations with other agents have shown better results. Paclitaxel combined with gemcitabine, ifosfamide, methotrexate or cisplatin showed a 15–60% response rate and taxanes combined with ifosfamide a 15–25% response rate (Tu *et al*, 1995; McCaffrey *et al*, 1997; Witte *et al*, 1997; Sweeney *et al*, 1999; Krege *et al*, 2001; Meluch *et al*, 2001; Sternberg *et al*, 2001; Bellmunt *et al*, 2002; Vaughn *et al*, 2002). Ifosfamide showed a 20% response rate in an ECOG study but an unacceptable frequency of grade 3–4 CNS and renal toxicities were noted (Witte *et al*, 1997). Toxicity remains the major limiting aspect of these regimens (Roth *et al*, 1994; Dreicer *et al*, 1996; Witte *et al*, 1997).

Vinflunine and pemetrexed as new single agents have been studied in phase II trials (Culine *et al*, 2006; Sweeney *et al*, 2006). Vinflunine showed an 18% response rate and a 67% disease control rate and pemetrexed, a new-generation antifolate, showed a favourable therapeutic index (an overall response rate of 27.7%). Incorporation of these new agents as second-line treatment combination regimens is expected in future studies.

Several regimens studied as second-line chemotherapy for urothelial carcinoma have variable response rates. This is likely due to the variability in drug activity as well as the different patient populations evaluated in different studies (Sweeney *et al*, 2006). In most studies, the regimens used for first-line chemotherapy were heterogeneous in a given study population (Tu *et al*, 1995). Most studies on second-line chemotherapy include patients who received M-VAC-, CMV-, GC- or taxane-based regimens, as initial chemotherapy, or patients who did not receive any chemotherapy (Tu *et al*, 1995; McCaffrey *et al*, 1997; Witte *et al*, 1997; Sweeney *et al*, 1999; Krege *et al*, 2001; Meluch *et al*, 2001; Bellmunt *et al*, 2002; Vaughn *et al*, 2002; Culine *et al*, 2006). These confounding factors make it difficult to interpret the true efficacy of a second-line chemotherapy regimen. In our study, all patients received the same regimen as first-line chemotherapy.

In conclusion, this is the first report on the efficacy and toxicity of M-VAC as second-line chemotherapy in patients who failed first-line GC. The main limitation of this study was the small number of cases evaluated. M-VAC showed encouraging efficacy and reversible toxicities in patients who had disease progression after GC chemotherapy. Therefore, M-VAC appears to be a reasonable option for patients who initially responded to first-line GC chemotherapy. There are few randomised trials on second-line chemotherapy for urothelial carcinoma; therefore, large randomised controlled studies are required to confirm the findings reported here.

## Figures and Tables

**Table 1:** Characteristics of the study population

**Characteristics**	**No. of patients**
Total enrolled patients	30
*Age*	
Median (years)	64
Range (years)	38–79
	
*Gender*	
Males	24
Females	6
	
*ECOG performance status*	
0	16
1	11
2	3
	
*Disease extents*	
Visceral metastases	19
Regional lymph-node metastases only	11
	
*Sites of diseases*	
Primary	14
Local recurrence	3
Lymph nodes	22
Lung	7
Liver	4
Bone	5
Others	2

**Table 2:** Toxicities

	**Grade 1**		**Grade 2**		**Grade 3**		**Grade 4**	
	**No.**	**%**	**No.**	**%**	**No.**	**%**	**No.**	**%**
Neutropaenia	1	3.3	2	6.7	6	20.0	13	43.3
Thrombocytopaenia	5	16.7	1	3.3	3	10.0	6	20.0
Anaemia	9	30.0	3	10.0	4	13.3	1	3.3
Mucositis	1	3.3	3	10.0	4	13.3	—	—
Alopecia	13	43.3	14	46.7				
Nausea/vomiting	14	46.7	8	26.7	—	—	—	—
Anorexia	10	33.3	5	16.7	1	3.3	—	—
Diarrhoea	2	6.7	1	3.3	—	—	—	—
Constipation	3	10.0	1	3.3	—	—	—	
Fatigue	5	16.7	1	3.3	—	—	—	—
Asthenia	5	16.7	2	6.7	—	—	—	—
Fever	3	10.0	1	3.3	—	—	—	—
Myalgia	5	16.7	1	3.3	—	—	—	—
Infection	—	—	—	—	1	3.3	—	—
Allergic reactions	1	3.3	1	3.3	—	—	—	—
Dizziness	1	3.3	—	—	—	—	—	—

**Table 3:** Characteristics of responders to second-line M-VAC regimen

**No.**	**Age**	**Site of disease**	**Response to GC**	**Treatment-free interval (month)**	**Response to M-VAC**	**RD (month)**	**PFS (month)**	**Current status**	**Survival (month)**
1	46	Bone Supraclavicular LN	PR	3.2	PR	6.6	8.6	Death	11.7
2	54	Retroperitoneal LN	PR	4.5	PR	3.3	6.4	Death	9.2
3	66	Bladder Lung Retroperitoneal LN	PR	5.7	PR	3.7	7.2	Alive	9.2+
4	72	Lung Retroperitoneal LN	PR	3	PR	3.9	7.6	Death	10.9
5	69	Pelvic recurrence Lung Retroperitoneal LN	PR	2	PR	3.8	7.1	Death	10.6
6	69	Bladder Retroperitoneal LN	PD	7	CR	1.5	4.4+	Alive	5.8+
7	64	Retroperitoneal LN Carcinomatosis	CR	1	PR	1.5	2.5+	Alive	5.3+
8	79	Retroperitoneal LN	CR	10.4	CR	7.2	10.6+	Alive	11.6+
9	52	Bladder Scrotum	PD	2.5	PR	3.9	6.7	Death	15.8

Abbreviations: CR=complete response; LN=lymph node; PD=progressive disease; PFS=progression-free survival; PR=partial response; RD=response duration.
